# miR-181a-2-3p Stimulates Gastric Cancer Progression *via* Targeting MYLK

**DOI:** 10.3389/fbioe.2021.687915

**Published:** 2021-10-18

**Authors:** Jianjie Li, Xiaoyue Xu, Chunhui Liu, Xiaoxue Xi, Yang Wang, Xiaotang Wu, Hua Li

**Affiliations:** ^1^ Department of Gastrointestinal Surgery, Tangshan Central Hospital, Tangshan, China; ^2^ Department of Gastrointestinal Surgery, Tangshan Gongren Hospital, Tangshan, China; ^3^ Department of General Surgery, North China University of Science and Technology Affiliated Hospital, Tangshan, China; ^4^ Shanghai Engineering Research Center of Pharmaceutical Translation, Shanghai, China

**Keywords:** metastasis, proliferation, gastric cancer, MYLK, mir-181a-2-3p

## Abstract

**Background:** The abnormal expression of miRNAs facilitates tumorigenesis and development. miR-181a-2-3p is up-regulated in various cancers, yet its mechanism in gastric cancer (GC) remains elusive.

**Objective:** To understand mechanism of miR-181a-2-3p stimulating GC cell progression *via* targeting Myosin Light Chain Kinase (MYLK) expression.

**Methods:** Downstream genes of miRNA of interest were predicted in TargetScan and miRTarBase. qRT-PCR and western blot were applied to assess miR-181a-2-3p and MYLK expression in GC cells and normal cells. Dual-luciferase and RIP assays were completed to assess binding of miR-181a-2-3p and MYLK. Cell Counting Kit-8 (CCK-8) assay was conducted for detecting viability of AGS and SNU-1 cells, while Transwell tested migratory and invasive abilities of cells. Nude mouse transplantation tumor experiment was performed to assay tumor growth *in vivo*.

**Results:** miR-181a-2-3p was notably increased in human GC cell lines, while MYLK was remarkably down-regulated. RIP and dual-luciferase assay disclosed that miR-181a-2-3p targeted MYLK and repressed MYLK. Forced miR-181a-2-3p expression fostered GC cell proliferation, invasion, migration, and fostered tumor growth *in vivo*. Promoting effect of miR-181a-2-3p on GC cells was reversed when miR-181a-2-3p and MYLK were simultaneously overexpressed.

**Conclusion:** miR-181a-2-3p facilitated GC cell progression by targeting MYLK, and it may be a pivotal prognostic biomarker in investigating molecular mechanism of GC.

## Introduction

Gastric cancer (GC) is a frequent malignancy with a low survival rate (Smyth et al., 2020). In 2018, more than 1 million new cases of GC were reported with 783,000 deaths. The mortality of GC ranks second among all malignant tumors in China (Zhao et al., 2020), thus putting forward higher requirements for the early prevention and diagnosis of GC. A growing body of methods are adopted for management of GC, whereas the recovery rate of patients is still low (Wei et al., 2019). Therefore, detecting and identifying novel biomarkers is crucial to the treatment of GC.

Research has evinced that miRNAs are associated with regulatory processes of promoting or inhibiting cancer cells by serving as biomarkers for management ofcancers (Lee and Dutta, 2009; Mishra et al., 2016; Liu et al., 2018). miRNAs can precisely regulate the activation and differentiation of cells, and their dysregulation becomes a potential cause of autoimmune diseases, with the fact that various miRNAs have changed in patients with autoimmune diseases (van den Hoogen et al., 2018). Moreover, miRNAs act as modulators in diverse biological processes like cell differentiation, proliferation, metabolism, and cancer development (Shin and Chu, 2014; Khan et al., 2019). An increasing number of studies substantiated that miRNAs are capable of regulating the downstream target genes, and thus play an oncogenic or tumor-suppressive role in GC (Wei and Gao, 2019). The forced miR-18a-3p expression and miR-4286 can repress BZRAP1 to enhance proliferation and movement of GC cells, thus leading to tumor progression *in vitro* (Liu et al., 2019; Tsai et al., 2020), whereas miR-320a suppresses the progression of GC by targeting PBX3 (Li et al., 2019). Therefore, it is essential to understand function of miRNAs in GC.

miR-181a-2-3p belongs to miR-181 family (Ji et al., 2009). A study uncovered that it is noticeably reduced in tumor stem cells and its exogenous expression in cervical cancer suppresses SOX2, thereby reducing bulk of tumor stem cells (Chhabra, 2018). It is found to be implicated in unfavorable prognosis of follicular variant of papillary thyroid carcinoma patients by Dettmer et al. (2013). Up to now, there is no research concerning miR-181a-2-3p in GC.

MYLK is a type of MLCK that induces the phosphorylation of Ser-19 of myosin ⅱ in smooth muscle contraction (Driska et al., 1981; Malencik et al., 1982). Paul et al. (2020) proved that MYLK mutated in metastatic breast cancer prior to primary breast cancer. Liu et al. (2020) identified three core genes (including MYLK) related to prostate cancer by bioinformatics analysis, and MYLK is considered as a promising diagnostic marker. MYLK, a downregulated gene in non-small cell lung cancer tissue, may be related to cancer metastasis (Tan and Chen, 2014). MYLK is a potential diagnostic or prognostic marker. Nonetheless, most studies are limited within the field of bioinformatics predictions, and few studies are about the relationship between MYLK and GC.

miR-181a-2-3p was a pivotal player in GC progression. We also found that miR-181a-2-3p had a great potential to promote GC epithelial cell proliferation, invasion, and migration *via* modulating MYLK expression. In sum, learning more about the effect and regulatory mechanism of miR-181a-2-3p on GC cell proliferation, invasion, and migration can provide us with insight into the solution of GC-related clinical problems.

## Materials and Methods

### Cell Cultivation

Human GC cell lines SNU-1 (ATCC^®^ CRL-5971™), AGS (ATCC^®^ CRL-1739), SNU-5 (ATCC^®^ CRL-5973™) were accessed from American Type Culture Collection (ATCC; Manassas, VA, United States), and human normal gastric epithelial cell line GES-1 (CBP60512) was accessed from COBIOER Co., Ltd. (Nanjing, China). A humidified incubator (37°C, 5% CO_2_) was recommended for cell maintenance. GES-1 cell line was prepared in Dulbecco’s Modified Eagle’s Medium (DMEM) plus 10% fetal bovine serum (FBS). AGS cell line was cultured in F-12K Medium (ATCC 30-2004) plus 10% FBS. SNU-1 cell line was prepared in Roswell Park Memorial Institute-1640 Medium (RPMI-1640; ATCC 30-2001) with 10% FBS. SNU-5 cell line was nurtured in Iscove’s Modified Dulbecco’s Medium (Catalog No. 30-2005) with 20% FBS.

### Cell Transfection

miR-181a-2-3p inhibitor, miR-181a-2-3p mimic, si-MYLK and corresponding negative controls (NC) were all accessed from GenePharma (Shanghai, China), and they were uniformly named as gene segments in the following text. After MYLK cDNA was amplified by PCR, product was inserted into the pcDNA3.1 vector with NHE I restriction endonuclease (# VT1001, Youbao Bio) to obtain MYLK overexpression vector (oe-MYLK). Then, cells (1 × 10^6^ cells/well) were seeded into 6-well plates and transfected with 50 nM gene fragment or 1 μg plasmid using Lipofectamine^®^ 2000 kit (Invitrogen; Thermo Fisher Scientific, Inc.) when cells reached 50% confluency. After transfection, cells were constantly cultured under standard conditions, and mediums were replaced 6 h later. Transfected cells were harvested 48 h later. The miRNA mimic/inhibitor and si-MYLK primer sequences used are: miR-181a-2-3p mimic: 5′-ACC​ACU​GAC​CGU​UGA​CUG​UAC​C-3’; mimic NC: 5′-ACU​CUA​UCU​GCA​CGC​UGA​CUU-3’; miR-181a-2-3p inhibitor: 5′-GGU​ACA​GUC​AAC​GGU​CAG​UGG​U-3’; inhibitor NC: 5′-CAG​UAC​UUU​UGU​GUA​GUA​CAA-3’. si-MYLK: 5′- UAA​UUU​UGA​AGA​AUC​CUU​CCC-3’; si-NC: 5′-GCG​UUC​AAC​UAG​CAG​ACC​A-3’.

### qRT-Polumerase Chain Reaction

Ten ng total RNA extracted from cells by Trizol (Invitrogen, NY) reagent was subject to reverse transcription into cDNA with TaqMan^®^ MicroRNA Reverse Transcription Kit and stem-loop primers. With cDNA as a template, qRT-PCR was completed with TaqMan MicroRNA Assay using TaqMan^®^ universal PCR Master Mix: 95°C 2 min, 45 cycles of 95°C 15 s and 60°C 45 s, and then 72°C 45 s miR-181a-2-3p expression was standardized with U6 miRNA as an endogenous reference.

cDNA of mRNA was synthesized with High-Capacity cDNA Reverse Transcription Kit (Applied Biosystems, United States). qRT-PCR was completed according to TaqMan Gene Expression Assays protocol (Applied Biosystems, United States). GAPDH was treated as an endogenous control. PCR was completed: 95°C 10 min, 35 cycles of 95°C 15 s and 60°C 30 s, and then 72°C 45 s qRT-PCR was run in triplicate. The primer sequences were purchased from TAKARA company (Beijing, China) as presented in Table 1. Relative quantitative method (2^-△△CT^) was for calculating relative transcription levels of target genes (Xie et al., 2019).

**TABLE 1:** Primer sequences in qRT-RCR.

Primer sequence	Forward (5’→3′)	Reverse (5’→3′)
MYLK	CGA​CGA​GGC​ATT​CGA​TGA​GA	AGT​TTT​TCT​GCA​TTG​AGC​GGG
miR-181a-2-3p	ACA​CTC​CAG​CTG​GGA​ACA​TTC​AAC​GCT​GTC	GTG​TCG​TGG​AGT​CGG​CAA​TTC
U6	CTC​GCT​TCG​GCA​GCA​CAT​A	AAC​GAT​TCA​CGA​ATT​TGC​GT
GAPDH	GCC​GCA​TCT​TCT​TTT​GCG​TC	TAC​GAC​CAA​ATC​CGT​TGA​CTC​C

### Western Blot

Total protein extraction from cells in different groups was completed. BCA kit (Thermo, United States) was recommended for concentration assessment. After 30 μg samples were electrophoresed at 80 V for 35 min and 120 V for 45 min, they were transferred onto polyvinylidene fluoride (PVDF; Amersham, United States) membranes. Later, membranes were cultivated with rabbit anti-MYLK antibody (ab155506) and GAPDH (ab181602) at 4°C overnight after 1 h of blockage under standard conditions. After rinsed with PBST (phosphate buffered saline buffer + 0.1% Tween-20) 3 × 10 min each, horseradish peroxidase-labeled goat anti-rabbit IgG H&L (HRP) secondary antibody (ab6721) was supplemented for 1 h of incubation. PBST was utilized to rinse membranes 3 × 10 min each. Bands were imaged by an optical luminometer (GE, GeneQuant 1,300, United States) and intensity was detected by Image Pro Plus 6.0 (Media Cybernetics, United States). Antibodies were from abcam (Cambridge, United Kingdom).

### Cell Counting Kit-8

Transfected cells were digested and re-suspended, and then planted into 96-well plates (1 × 10^5^ cells/ml) with a density of 100 μl/well, followed by conventional incubation overnight. Cell viability was assayed with CCK-8 kit (Beyotime, China). 0, 24, 48 and 72 h later, 10 μl CCK-8 solution was supplemented to wells for consequent 4 h of cultivation. The absorbance at 450 nm was finally assayed with an enzyme-labeled instrument (BioTek Instruments, Inc., United States, Epoch microplate reader), and growth curves were plotted.

### Migration and Invasion Assays

Migration and invasion assays were carried out according to the procedures in previous studies (Zhang et al., 2019). The 8-μm pores Transwell chambers (Costar/Corning, MA) were used. In migration assay, 200 μL serum-free mediums suspending 5 × 10^4^ cells were added to upper chamber without Matrigel. For invasion assay, 200 μL serum-free mediums resuspending 1 × 10^5^ cells were placed in upper chamber with Matrigel (BD Biosciences). Both in invasion and migration assays, lower chambers were added with mediums containing 10% FBS as inducer. After being incubated for 24 h, methanol was utilized for fixing cells on lower surface and 0.1% crystal violet for staining. Randomly, 5 fields were chosen and cells were counted under a microscope (100x).

### Ribo Nucliec Acid Binding Protein Immunoprecipitation

Binding relationship of miR-181a-2-3p and MYLK was assayed with Magna RIP Kit (Millipore, Billerica, Massachusetts). After cells were rinsed in pre-cooled PBS and the supernatant was removed, cells were in ice bath for 5 min with lysis buffer (equal volume, P0013B, Beyotime) and followed by 10 min centrifugation at 14,000 rpm at 4°C. Then, supernatant was collected. Part of cell extracts was taken as Input, while the rest was used for co-immunoprecipitation with antibodies. Specific steps were: In every co-immunoprecipitation reaction system, 50 μl cell extracts were rinsed with magnetic beads and re-suspended in RIP Wash Buffer (100 μl), and according to grouping, 5 μg antibody was supplemented for binding. Magnetic bead-antibody complexes were rinsed and re-suspended in RIP Wash Buffer (900 μl), and sequentially incubated with 100 μl cell extracts overnight at 4°C. Samples were placed onto a magnetic base to harvest magnetic bead-protein complexes. Antibodies used in RIP included rabbit polyclonal antibody Ago2 (ab32381, 1:50, Abcam, United Kingdom) mixing for 30 min and monoclonal antibody IgG (1:100, ab172730, Abcam, United Kingdom), the NC. Proteinase K was used for digesting samples and qRT-PCR assessed MYLK mRNA level.

### Dual-Luciferase Reporter Gene Assay

MYLK sequence was amplified from cDNA, ligated to the pmirGLO (Promega, WI, United States) luciferase reporter plasmid for wild-type MYLK (MYLK-WT) establishment. Primer sequences are: MYLK-Forward primer: 5′ CTT​GAG​GCT​GTT​GCT​GAG​GA 3’; Reverse primer: 5′ TCT​ATC​TGG​AAG​TGG​CGG​GA 3’. Mutant MYLK (MYLK-MUT) was built by PCR site-directed mutagenesis kit (Beyotime, #D0206). Inhibitor NC, miR-181a-2-3p inhibitor, mimic NC, miR-181a-2-3p mimic were co-transfected with MYLK-WT or MYLK-MUT into AGS and SNU-1 cells, respectively. Dual-Luciferase Reporter Assay System (Promega, United States) was utilized for luciferase activity detection.

### Nude Mouse Transplantation Tumor Experiment

Four-week-BALB/c nude mice from Kunming Institute of Zoology (KIZ) center were used. All animal experiments were conducted follwing the Guide for the Care and Use of Laboratory Animals and approved by animal ethics committee. AGS Cells were incubated with 200 nM miR-181a-2-3p agomir or agomir NC for 2 days. Then the transfected cells were collected and digested with trypsin into cell suspension at a concentration of 1.0 × 10^7^ cells/mL. Six nude mice were randomly divided into two groups (n = 3/group). 200 μL cell suspension was inoculated into the subcutaneous tissue of the right lower extremity of nude mice to construct the xenograft tumor model. Twelve days after inoculation, intratumor injections of miR-181a-2-3p agomir or agomir NC were given every 6 days at 10 nmol each time. Tumor volume was detected every 6 days. The volume is measured using a vernier caliper (V = a * b (Zhao et al., 2020)/2; a: tumor length; b: tumor short diameter). After 30 days later, tumors from sacrificed mice were excised and weighed.

### Statistical Analysis

Data were handled with SPSS 21.0 (SPSS, Inc., Chicago, IL, United States). All experiments were completed in 3 replicates. Measurement data were presented in means ± standard deviation, and *t* test was adopted for the comparison of two groups. *p* < 0.05 meant statistically significant.

## Results

### miR-181a-2-3p is Highly Expressed in GC Cells

**FIGURE 1:**
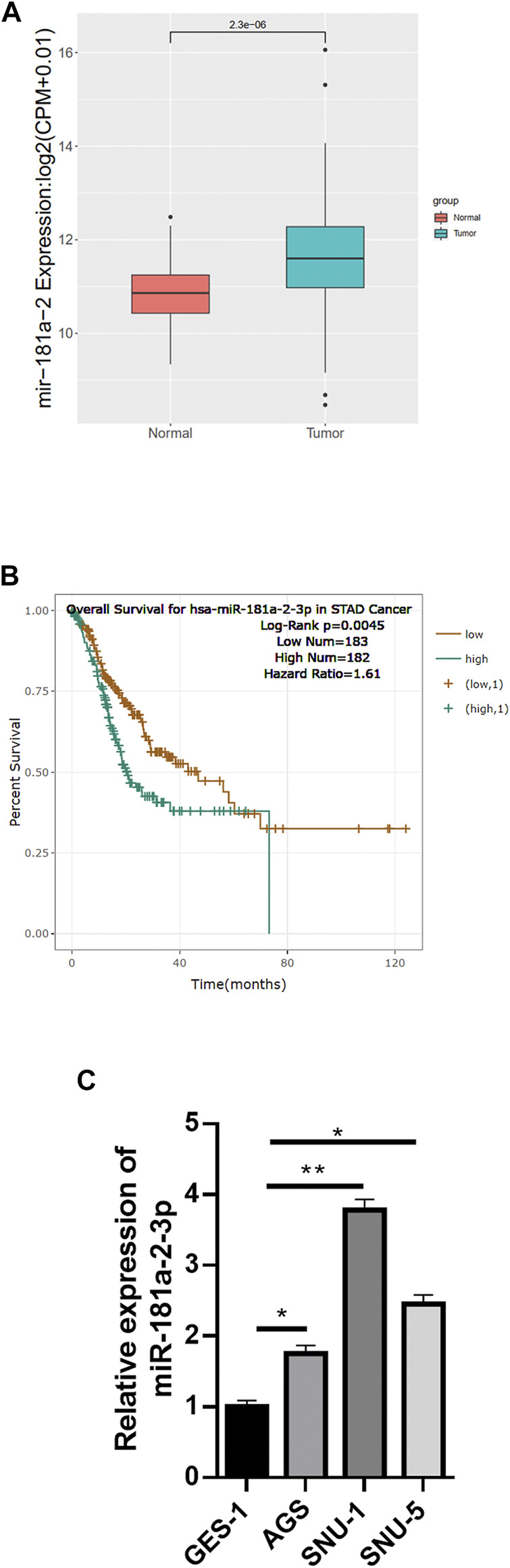
miR-181a-2-3p is up-regulated in GC cells. **(A)** The differential expression of miR-181a-2 in TCGA-STAD dataset (red: normal, blue: tumor); **(B)** Overall survival curves of patients with high/low miR-181a-2-3p expression (grouping according to median expression of miR-181a-2-3p in TCGA); **(C)** qRT-PCR measured miR-181a-2-3p expression in GES-1, AGS, SNU-1, SNU-5 cells; *p < 0.05, **p < 0.01.

Referring to data from TCGA-STAD (https://portal.gdc.cancer.gov/), miR-181a-2-3p was upregulated prominently in GC tissue relative to normal tissue (Figure 1A). Survival analysis suggested that high miR-181a-2-3p expression was linked with unfavorable prognosis of patients (Figure 1B). Literature reported that miR-181a-2-3p is highly expressed in adenoid cystic carcinoma (ACC), and patients with high miR-181a-2-3p expression have a relatively low survival rate (Andreasen et al., 2018). miR-181a-2-3p can also serve as a biomarker for prognosis of head and neck cancer (Yoke-Kqueen et al., 2011). Since miR-181a-2-3p has rarely studied in GC previously, we chose miR-181a-2-3p as study object. miR-181a-2-3p level in GES-1, AGS, SNU-1, SNU-5 cells were assayed by qRT-PCR. miR-181a-2-3p was differentially highly expressed in GC cell lines relative to that in GES-1 cells (Figure 1C). Therefore, AGS with relatively low miR-181a-2-3p and SNU-1 with highest miR-181a-2-3p expression were selected for subsequent experiments.

## Overexpressing miR-181a-2-3p Promotes GC Cell Malignant Progression, While Silencing miR-181a-2-3p Exerts an Opposite Effect

**FIGURE 2:**
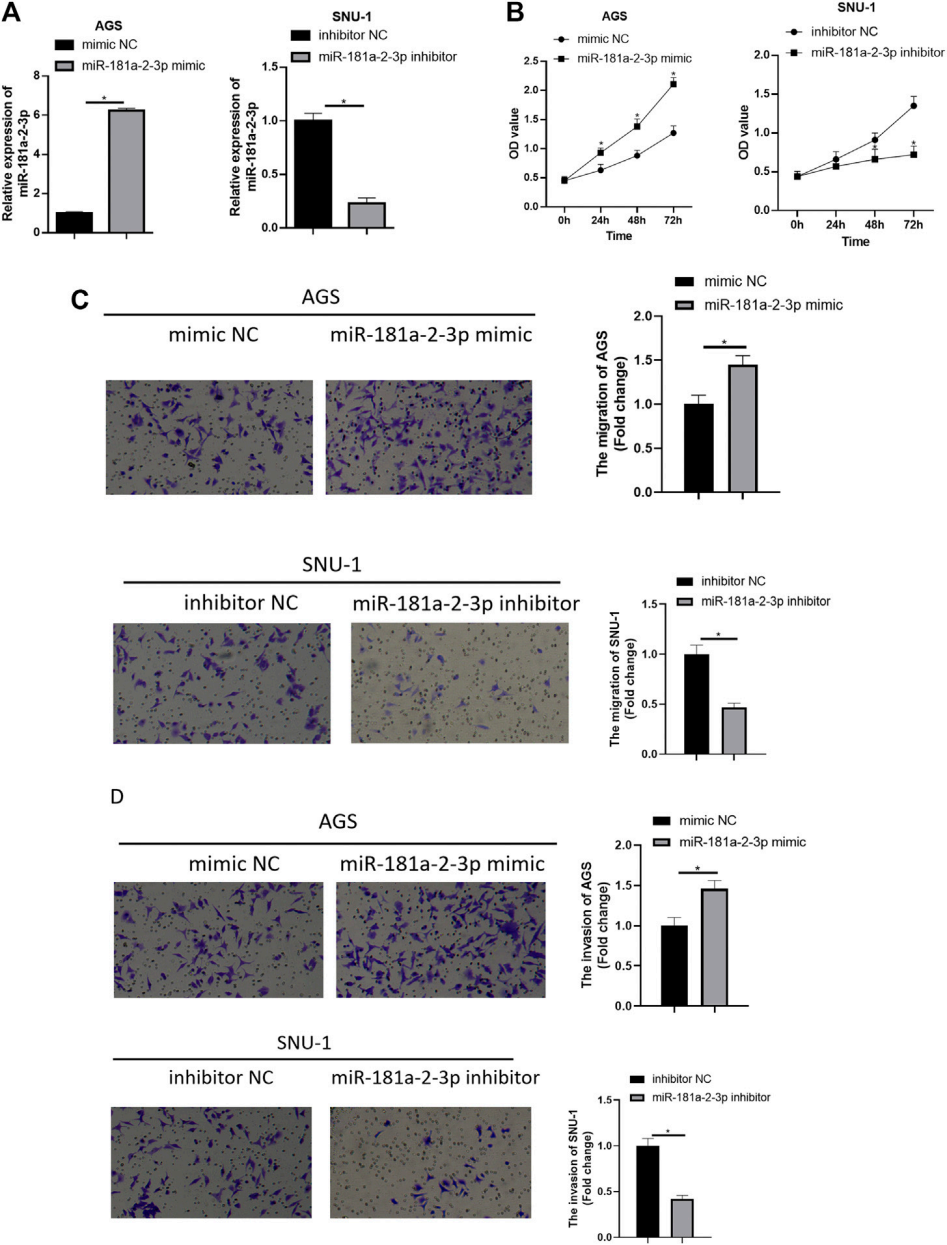
Overexpressing miR-181a-2-3p promotes GC cell progression while silencing miR-181a-2-3p exerts an opposite effect. **(A)** qRT-PCR assayed miR-181a-2-3p in SNU-1 and AGS cells with varying treatments; CCK-8 and Transwell assays (100×) were performed to measure **(B)** cell viability, **(C)** cell migration and **(D)** invasion of AGS and SNU-1 cells; *p < 0.05.

To further probe impact of miR-181a-2-3p on GC cells, miR-181a-2-3p was overexpressed in AGS cell line, and miR-181a-2-3p inhibitor was transfected into SNU-1 cells. As determined by qRT-PCR in AGS cells, miR-181a-2-3p level in miR-181a-2-3p mimic group was higher than mimic NC group, and results in SNU-1 cells were as expected (Figure 2A). As depicted in Figure 2B, CCK-8 manifested that forced miR-181a-2-3p expression markedly increased AGS cell viability, silencing miR-181a-2-3p conspicuously hindered SNU-1 cell viability. Transwell measured effect of overexpressed miR-181a-2-3p on AGS or SNU-1 cell migration and invasion abilities, showing that these abilities of AGS cells were considerably elevated with the forced expression of miR-181a-2-3p. While SNU-1 cell migration and invasion potentials were restrained in miR-181a-2-3p inhibitor group (Figures 2C,D). The findings elucidated that up-regulation of miR-181a-2-3p stimulated GC cell proliferation, invasion and migration while silencing miR-181a-2-3p restrained their abilities.

### miR-181a-2-3p Targets and Inhibits Myosin Light Chain Kinase Expression

**FIGURE 3:**
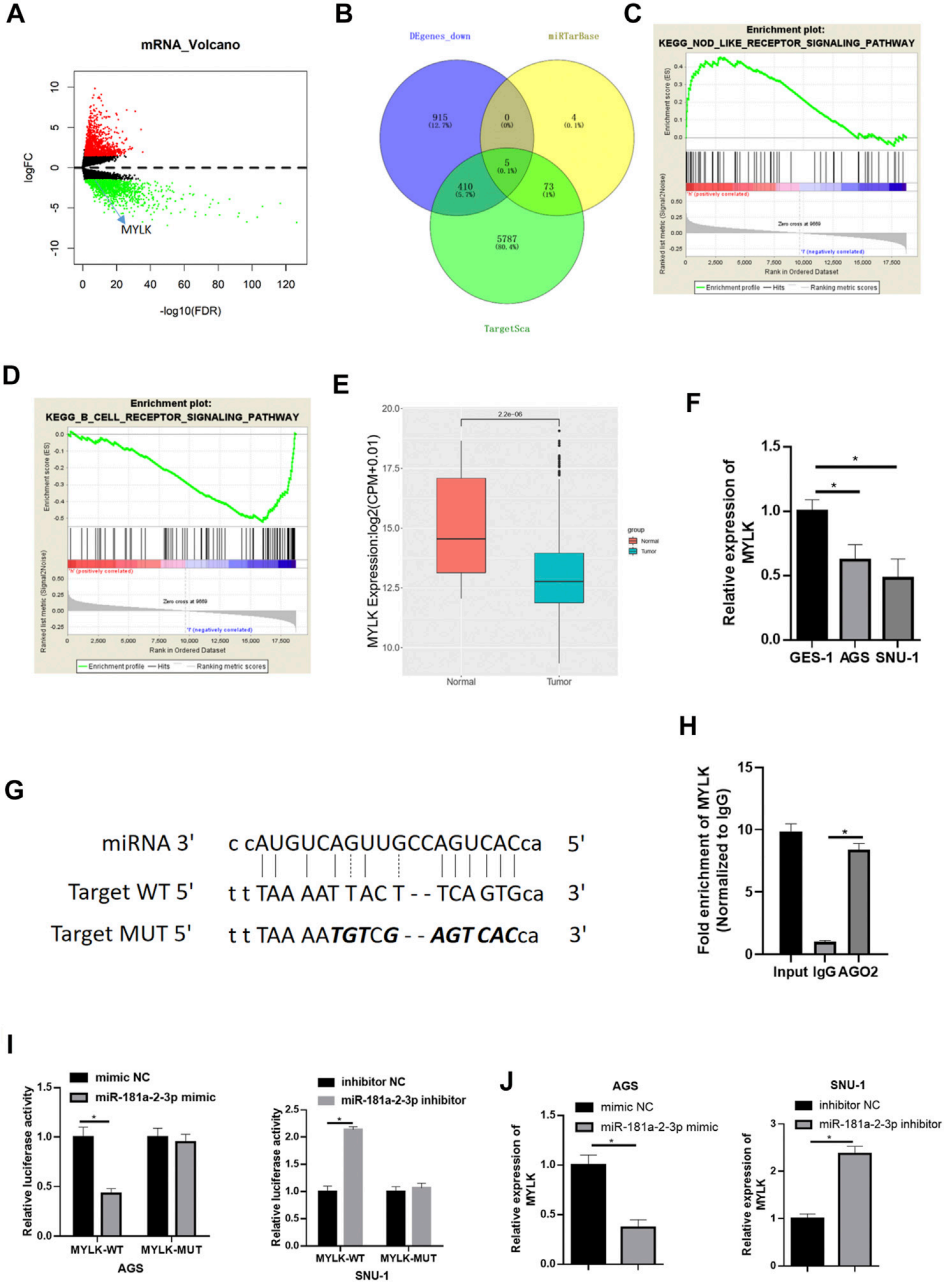
miR-181a-2-3p targets and inhibits MYLK **(A)** Volcano plot of differential mRNAs in TCGA-STAD dataset (red: up-regulated, green: down-regulated); **(B)** The intersection of the down-regulated DEmRNAs in TCGA and genes predicted by TargetScan and miRTarBase; The most enriched GSEA pathways of **(C)** high MKLK expression and **(D)** low MKLK expression; **(E)** Differential MYLK in TCGA-STAD (red: normal, blue: tumor); (F) qRT-PCR assayed MYLK in GES-1, AGS, SNU-1; **(G)** The binding sites of miR-181a-2-3p and MYLK (the italic bold was mutation sites, top: miRNA sequence, middle: wt target DNA sequence of MYLK, bottom mutated target sequence of MYLK); **(H)** RIP experiments assessed binding relationship of miR-181a-2-3p and MYLK, and the binding relationship was validated by **(I)** Dual-luciferase reporter gene assay; **(J)** qRT-PCR was conducted for the determination of MYLK expression; *p < 0.05.

To learn more about downstream modulatory mechanism of miR-181a-2-3p, miRTarBase (http://mirtarbase.mbc.nctu.edu.tw/php/index.php) and TargetScan (http://www.targetscan.org/vert_72/) were used to predict downstream targets of miR-181a-2-3p. Meanwhile, differential analysis was conducted on mRNAs from TCGA-STAD (Figure 3A). Five candidate genes (MYLK, ABCG2, NSG2, TNFRSF13B, PSD) were obtained by intersecting down-regulated genes in TCGA-STAD dataset with predicted genes (Figure 3B). Literature reported that androgen receptor (AR) promotes GC cell invasion and metastasis by regulating Myosin light chain kinase (MYLK) (Xia et al., 2019). Besides, it is theorized that MYLK regulates the invasion and metastasis of some malignancies by catalyzing the phosphorylation of myosin light chains (MLC) (Lin et al., 2018). Hence, we reasoned that miR-181a-2-3p facilitated GC epithelial cell proliferation, migration, and invasion *via* suppressing MYLK expression.

Gene Set Enrichment Analysis (GSEA) was carried out. It was found that high expression of MYLK was significantly enriched in KEGG_NOD_LIKE_RECEPTOR_SIGNALING_PATHWAY (Figure 3C), which was associated with the immune system, suggesting that high expression of MYLK promoted the progression of GC by activating the pathway (NOM p-val < 0.05). Besides, low-expression of MYLK was enriched in KEGG_B_CELL_RECEPTOR_SIGNALING_PATHWAY, and such pathway was related to B-cell immunoregulation (Figure 3D). Differential expression analysis was conducted on MYLK in TCGA-STAD, disclosing that MYLK was conspicuously lowly expressed in GC (Figure 3E). MYLK expression in cells was assayed *via* qRT-PCR. The result was congruous with result predicted from TCGA database (Figure 3F). These findings suggested low MYLK level in GC tissue and cells.

We predicted the binding sites of miR-181a-2-3p in MYLK by bioinformatics methods on website (http://mirtarbase.mbc.nctu.edu.tw/php/index.php) (Figure 3G) and further verified by RIP experiment (Figure 3H). Dual-luciferase assay denoted that overexpressing miR-181a-2-3p considerably lowered luciferase activity of AGS cells with MYLK-WT but had no marked influence on MYLK-MUT. Reversely, silencing miR-181a-2-3p conspicuously increased luciferase activity of MYLK-WT in SNU-1 cells but still had no marked influence on MYLK-MUT (Figure 3I). MYLK exhibited a significantly decreased expression level after miR-181a-2-3p was overexpressed in AGS cells, while its expression was forced when miR-181a-2-3p was silenced in SNU-1 cells (Figure 3J). Hence, miR-181a-2-3p hindered MYLK expression.

## Forced MYLK Expression Restrains GC Cell Malignant Progression, With the Opposite Effect by Knocking Down MYLK

**FIGURE 4:**
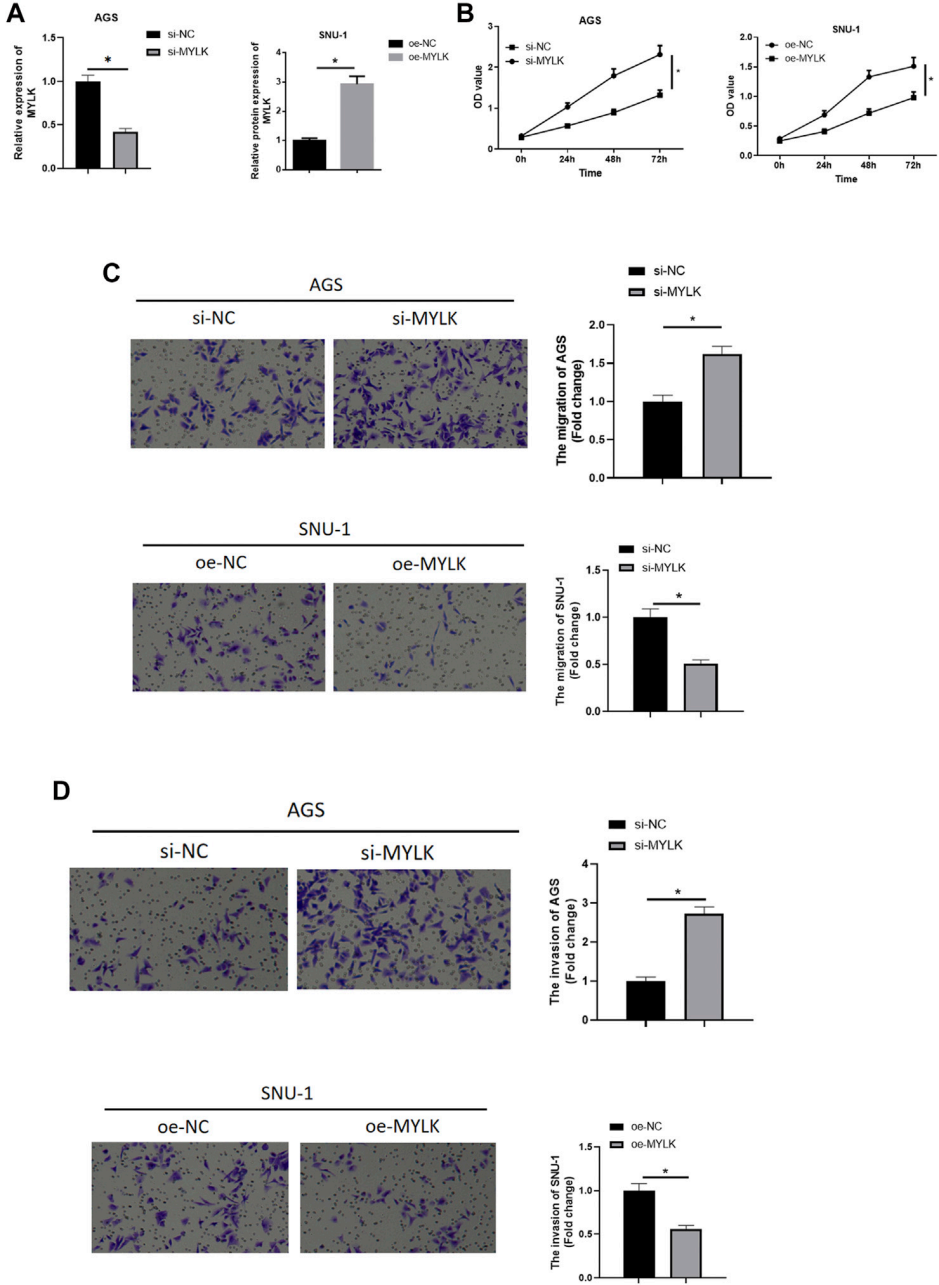
The forced MYLK expression restrains GC cell malignant progression, with the opposite effect by knocking down MYLK. **(A)** qRT-PCR assayed MYLK mRNA expression in AGS and SNU-1 cells with varying treatments; CCK-8 and Transwell assays (100×) were performed to measure **(B)** cell viability, **(C)** cell migration and **(D)** invasion of AGS and SNU-1 cells; *p < 0.05.

To validate whether MYLK is an essential participant in GC progression, MYLK was overexpressed/silenced in 2 cell lines. In AGS cells expressing si-MYLK, MYLK expression was decreased, while in SNU-1 cells expressing oe-MYLK, MYLK expression was increased, suggestive of favorable transfecting efficacy as determined by qRT-PCR (Figure 4A). Cell biological functions were assayed by CCK-8 and Transwell, and as depicted in Figures 4B–D, silencing MYLK upregulated proliferative, migratory, and invasive potentials of AGS cells, while overexpressing MYLK repressed these properties of SNU-1 cells. Hence, MYLK was a pivotal modulator in GC cell progression.

### miR-181a-2-3p Promotes GC Cell Progression by Suppressing Myosin Light Chain Kinase

**FIGURE 5:**
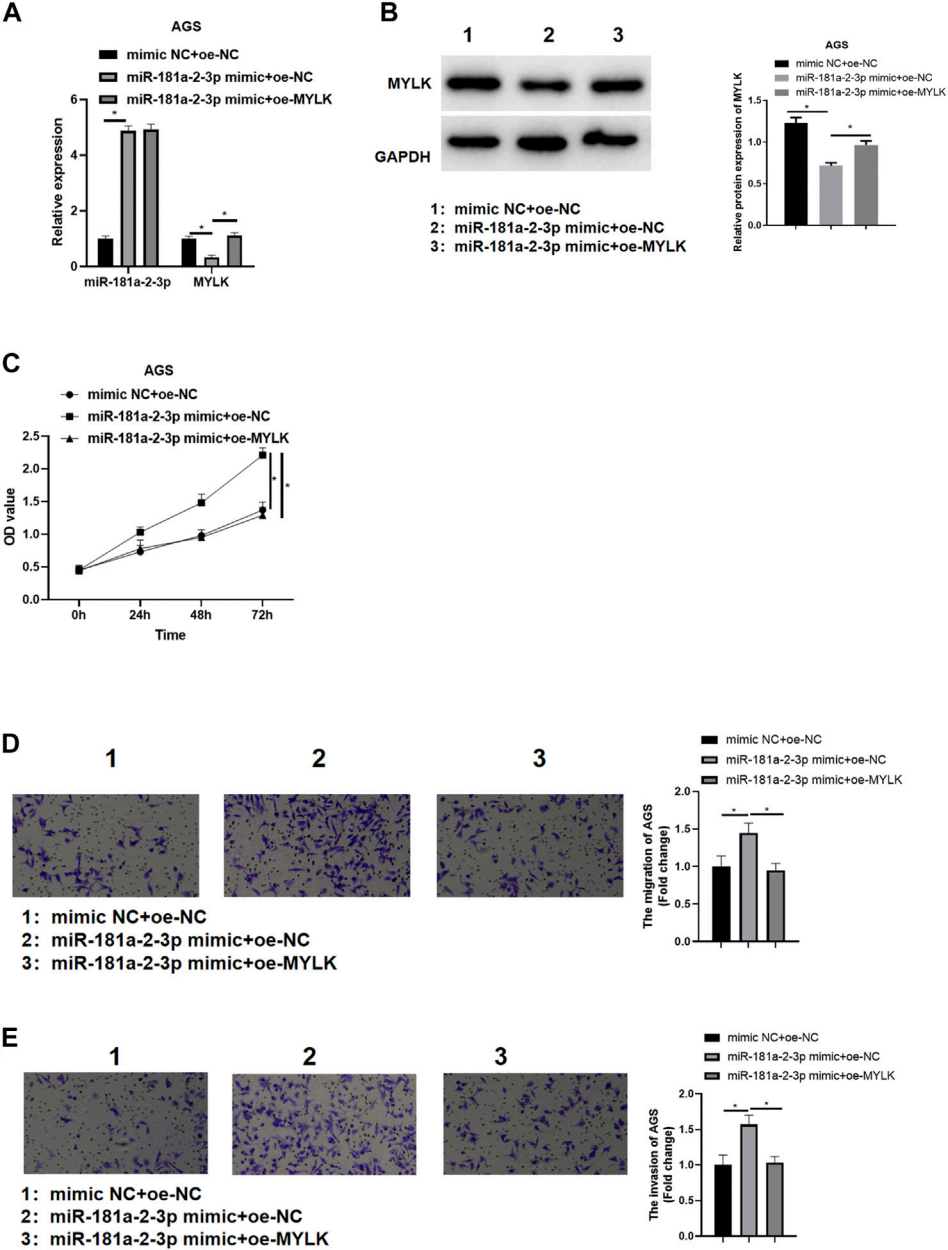
miR-181a-2-3p promotes GC cell progression *via* hampering MYLK miR-181a-2-3p and MYLK were simultaneously overexpressed in AGS cells; **(A)** qRT-PCR and **(B)** western blot examined miR-181a-2-3p and MYLK; CCK-8 and Transwell (100×) determined cell **(C)** viability, **(D)** migration and **(E)** invasion of AGS cells; *p < 0.05.

We confirmed that miR-181a-2-3p could suppress MYLK. miR-181a-2-3p and MYLK were simultaneously overexpressed to identify the impact of miR-181a-2-3p targeting MYLK on AGS cell proliferation, invasion, and migration. qRT-PCR and western blot were used to examine miR-181a-2-3p and MYLK, finding that miR-181a-2-3p was extremely highly expressed in miR-181a-2-3p mimic + oe-NC group in comparison to that in mimic NC + oe-NC, while MYLK showed a significantly decreased expression level. Besides, MYLK was observed to be remarkably expressed in miR-181a-2-3p mimic + oe-MYLK group than that in miR-181a-2-3p mimic + oe-NC group (Figures 5A,B). CCK-8 and Transwell were conducted to assay GC cell viability, cell migratory and invasive abilities, showing that these abilities were noticeably elevated after miR-181a-2-3p was overexpressed, while its promoting impact on these abilities were reversed when miR-181a-2-3p and MYLK were simultaneously overexpressed (Figures 5C–E). It was manifested that the forced miR-181a-2-3p expression fostered GC cell progression by hindering MYLK.

### miR-181a-2-3p Facilitates Tumor Growth in Mice

**FIGURE 6:**
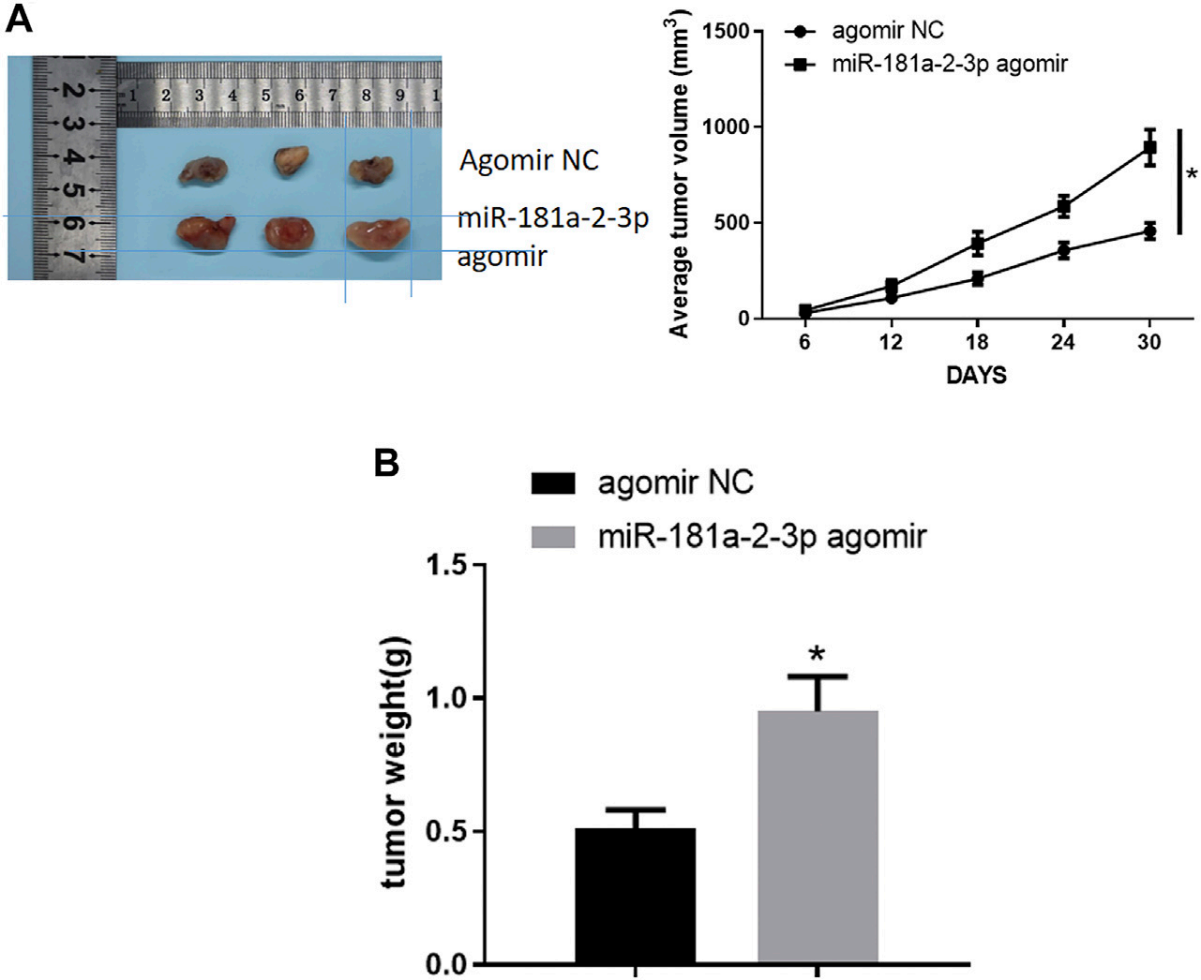
miR-181a-2-3p facilitates tumor growth *in vivo* **(A)** Volume of mouse tumors; **(B)** Weight of mouse tumors; *p < 0.05.

Nude mouse transplantation tumor experiment was designed to probe the impact of miR-181a-2-3p on GC tumor growth *in vivo*. As displayed in Figure 6A, growth velocity and volume of tumors expressing miR-181a-2-3p agomir were faster and larger than those expressing agomir NC. After tumors were weighed, weight of mouse tumors in oe-miR-181a-2-3p group was found to be heavier than that of mice in control group (Figure 6B). Hence, miR-181a-2-3p could foster tumor growth *in vivo*.

## Discussion

miRNAs are abnormally expressed in GC and pivotal in occurrence and development (Kang et al., 2018; Maruyama et al., 2018; Chen et al., 2019; Wang et al., 2019). Identifying the biological function of miRNAs is conducive to the exploration of new biomarkers and GC progression.

miRNAs are identified important in multiple cellular processes, such as proliferation, differentiation, and cell apoptosis (Lu et al., 2005; Nikiforova et al., 2009). A study (Ni et al., 2019) showed that propofol hinders GC progression by the regulatory axis of miR-29/MMP-2. Chao Wei and Jian-Jun Gao et al. (Wei and Gao, 2019) found that down-regulation of miR-383–5p facilitates GC cell progression, being implicated in poor prognosis. miR-524–5p suppresses GC cell proliferation by regulating CASP3 (Zhu et al., 2019). Here, we obtained miRNA expression data of TCGA-STAD dataset from TCGA database, finding increased miR-181a-2-3p in tumor tissue, being associated with unfavorable prognosis of patients. miR-181a-2-3p exhibited a noticeably elevated expression level in human GC cell lines as revealed by qRT-PCR. miR-181a-2-3p high expression hastened GC cell proliferation, invasion, and migration, while their abilities were hampered when it was silenced. Besides, nude mouse transplantation tumor experiment manifested that miR-181a-2-3p fostered tumor growth *in vivo*.

We further investigated downstream regulatory mechanism of miR-181a-2-3p by predicting possible targets of miR-181a-2-3p. TargetScan and miRTarBase database predicted downstream targets of miR-181a-2-3p, and candidates were obtained by intersecting down-regulated DEmRNAs in TCGA database with predicted genes. MYLK was identified as downstream target of miR-181a-2-3p in combination with literature. In non-smooth muscle cells, MYLK participates in inflammatory diseases by regulating tight junctions and intestinal epithelium barrier functions (Eutamene et al., 2005; Shen et al., 2010). Aberrant MYLK is related to malignant transformation of normal cells and affects the tumor cell migration and invasion abilities (Cui et al., 2010; Chen et al., 2012). Additionally, MYLK is a key participant in cell progression of breast cancer (Cui et al., 2010), prostate cancer (Gu et al., 2006) and colon cancer (Han et al., 2011). A study also reported that hypermethylation of serum MYLK can be taken as a diagnostic marker for GC (Chen et al., 2012). We manifested that MYLK was extremely lowly expressed in GC cell lines, while miR-181a-2-3p hampered MYLK expression. Overexpressing MYLK rescued the promoting of miR-181a-2-3p on cell viability, cell migratory and invasive abilities, indicating that its up-regulation fostered GC cell progression through MYLK suppression.

In a word, miR-181a-2-3p is pivotal in GC progression *via* regulating MYLK expression, which provides a potential marker and a new molecular target for effective management of GC.

## Data Availability

The original contributions presented in the study are included in the article/Supplementary Material, further inquiries can be directed to the corresponding author.
